# The pedunculopontine tegmental nucleus and the nucleus basalis magnocellularis: Do both have a role in sustained attention?

**DOI:** 10.1186/1471-2202-9-16

**Published:** 2008-01-30

**Authors:** Claire L Rostron, Morag J Farquhar, Mary P Latimer, Philip Winn

**Affiliations:** 1 Life Sciences, The Open University, Walton Hall, Milton Keynes, MK7 6AA, UK; 2 School of Psychology, University of St Andrews, St Mary's College, South Street, St Andrews, KY16 9JU, UK

## Abstract

**Background:**

It is well established that nucleus basalis magnocellularis (NbM) lesions impair performance on tests of sustained attention. Previous work from this laboratory has also demonstrated that pedunculopontine tegmental nucleus (PPTg) lesioned rats make more omissions on a test of sustained attention, suggesting that it might also play a role in mediating this function. However, the results of the PPTg study were open to alternative interpretation. We aimed to resolve this by conducting a detailed analysis of the effects of damage to each brain region in the same sustained attention task used in our previous work. Rats were trained in the task before surgery and post-surgical testing examined performance in response to unpredictable light signals of 1500 ms and 4000 ms duration. Data for PPTg lesioned rats were compared to control rats, and rats with 192 IgG saporin infusions centred on the NbM. In addition to operant data, video data of rats' performance during the task were also analysed.

**Results:**

Both lesion groups omitted trials relative to controls but the effect was milder and transient in NbM rats. The number of omitted trials decreased in all groups when tested using the 4000 ms signal compared to the 1500 ms signal. This confirmed previous findings for PPTg lesioned rats. Detailed analysis revealed that the increase in omissions in PPTg rats was not a consequence of motor impairment. The video data (taken on selected days) showed reduced lever orientation in PPTg lesioned rats, coupled with an increase in unconditioned behaviours such as rearing and sniffing. In contrast NbM rats showed evidence of inadequate lever pressing.

**Conclusion:**

The question addressed here is whether the PPTg and NbM both have a role in sustained attention. Rats bearing lesions of either structure showed deficits in the test used. However, we conclude that the most parsimonious explanation for the deficit observed in PPTg rats is inadequate response organization, rather than impairment in sustained attention. Furthermore the impairment observed in NbM lesioned rats included lever pressing difficulties in addition to impaired sustained attention. Unfortunately we could not link these deficits directly to cholinergic neuronal loss.

## Background

Understanding the neural basis of attention remains a fundamental goal of neuroscience, of as much importance to studies of information processing as it is to clinical studies of the disorders in which attention is impaired. Research has focused on many brain structures, from the cerebral cortex to the deep brainstem, and on many neurotransmitters, though acetylcholine (ACh) and noradrenaline are the most frequently implicated ones. One structure that has been argued to be involved in attentional processing is the pedunculopontine tegmental nucleus (PPTg), part of the pontomesencephalic tegmentum [1-3]. It consists of cholinergic and non-cholinergic neurons bordered medially by the superior cerebral peduncle, rostrally by the substantia nigra, and caudally by the parabrachial nucleus. Early studies of PPTg function concentrated on motor processes and behavioural state control, but recent work has emphasized its psychological functions (see Winn [4,5]). In line with this we have demonstrated previously that there are severe deficits in the performance of PPTg lesioned rats on a sustained attention task [3], while others have shown impaired performance on the five choice serial reaction time task following PPTg damage [6].

There is good reason to suspect that an intact PPTg would be necessary for the successful performance of visual attention tasks on anatomical grounds. Firstly, electrical stimulation of PPTg can change the pattern of neocortical EEG from large amplitude, slow oscillations to low amplitude, high frequency activity consistent with cortical activation [7]. In addition, stimulation of PPTg also produces an increase in acetylcholine in the cortex [8]. The presence of ACh in the cortex increases the responsiveness of cortical neurons to excitatory input [9]. One route by which PPTg might achieve this influence over cortical activity is via its connections with the nucleus basalis magnocellularis (NbM) [10]. In intact rats the application of drugs to manipulate the excitability of ACh projections to cortex from the NbM produces correlated changes in the performance of attention tasks [11]. Furthermore, there is evidence that selective cholinergic depletion of NbM by 192 IgG saporin profoundly impairs sustained attention [12]. As a result, damage to the cholinergic neurons of the nucleus basalis magnocellularis represents an "ideal" benchmark against which to assess the functional impact of damage to the PPTg. Of course, if the NbM was the only route by which PPTg might influence the cortical processing of sensory information one could expect that the deficits produced following damage to each of these structures would be qualitatively similar (in much the same way as damage to the dorsal striatum produces performance changes in the 5 choice serial reaction time task that mirrors performance impairments seen following damage to the medial prefrontal cortex [13]).

However, in addition to connections with the NbM, all of the cholinergic neurons of the PPTg innervate thalamic nuclei (in fact the innervation of all other brain sites arises from collaterals of these connections [14]). The contact between cholinergic cells of PPTg and thalamic nuclei is made onto GABAergic inter-neurons, and it has been suggested that there is particularly dense innervation of visual thalamic nuclei [15]. The lateral geniculate nucleus (LGN) receives as much as 40% of its input from PPTg [16,17]. Uhlrich et al. [18] showed *in vivo *that stimulation of PPTg neurons projecting to LGN enhances the visual response of relay cells without changing the receptive field size, a mechanism that, according to these authors, would increase the resolution of visual perception.

There is also strong cholinergic innervation from PPTg to the GABAergic cells of the thalamic reticular nucleus (TRN), a structure previously shown to produce deficits in attention when it is damaged [19]. However, TRN also receives a portion of its cholinergic input (approximately two-thirds) from the NbM [20] suggesting possible concerted action of NbM ACh neurons and PPTg ACh neurons on this nucleus. Here the application of cholinergic agonists blocks spindle activity [21]. This is of relevance because spindles occur in the TRN during behavioural immobility, light sleep, and anaesthesia, suggesting that the cholinergic influence on TRN from both NbM and PPTg may function to heighten behavioural arousal. Likewise the release of ACh from PPTg terminals depolarises thalamocortical cells and switches their firing from rhythmic bursting to tonic activation, a function that is proposed to gate the flow of sensory information to the cortex [22-24].

**Figure 1:**
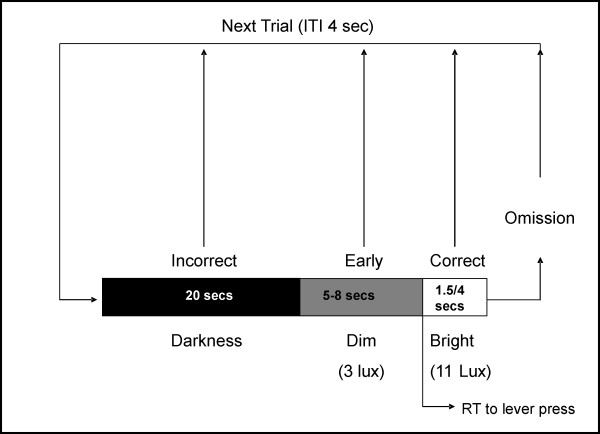
A schematic representation of the sustained attention task.

Previous behavioural work from this laboratory appears to support the anatomical evidence for PPTg involvement in attention. The primary effect of PPTg damage in a sustained attention task was an increase in the number of omissions, and a concomitant decrease in correct responses [3]. Furthermore, and perhaps crucially, this particular deficit was significantly reduced by lengthening the visual signal. Note that this manipulation is hypothesised to decrease the attentional load. Thus when demands on attention were reduced, PPTg lesioned rats improved their performance. The problem is that it is not entirely clear from these results alone *why *PPTg lesioned animals made such a high level of omissions. One hypothesis that cannot be dismissed is that PPTg lesioned rats suffered a simple motor impairment. This is a particularly pertinent point given early functional work implicating PPTg in locomotion. Unfortunately this alternative hypothesis exists because the visual attention task, developed to examine the effects of frontal lobe lesions [25,26], confounds both the motor and attention benefits of increasing the signal length: a correct response can only occur during the presentation of the visual signal. As a result, lengthening the visual signal also lengthens the time window allowed for making a response. Regrettably, the latency to make a correct response was not measured in our previous experiment. The current study therefore aimed to overcome this methodological limitation. We aimed also to examine, in detail, the behavioural effects of PPTg lesions in a sustained attention task (schema illustrated in Figure 1) by including analysis of video data. In particular we wanted to address the question: *why *do PPTg lesioned rats make more omissions? Is it the result of an inability to sustain attention? We also aimed to compare the behaviour of PPTg lesioned rats with that of rats with 192 IgG saporin lesions of the nucleus basalis magnocellularis (192 IgG saporin causes selective cholinergic cell depletion).

## Results

### Pre-surgery sustained attention data

One-way ANOVAs conducted on the mean sustained attention operant task data for all groups across the 3 criterion days prior to surgery (data not shown) showed no significant between group differences: for **percent correct responses**: F (_2,18_) = 0.67, p = 0.52; for **signal omissions**: F (_2,18_) = 0.09, p = 0.92; for **percent early responses**: F (_2,18_) = 0.42, p = 0.66; for **percent dark responses**: F (_2,18_) = 3.13, p = 0.07; for **latency to lever press**: F (_2,18_) = 0.35, p = 0.71.

### Post-surgery sustained attention data: Days 1–10

**Figure 2:**
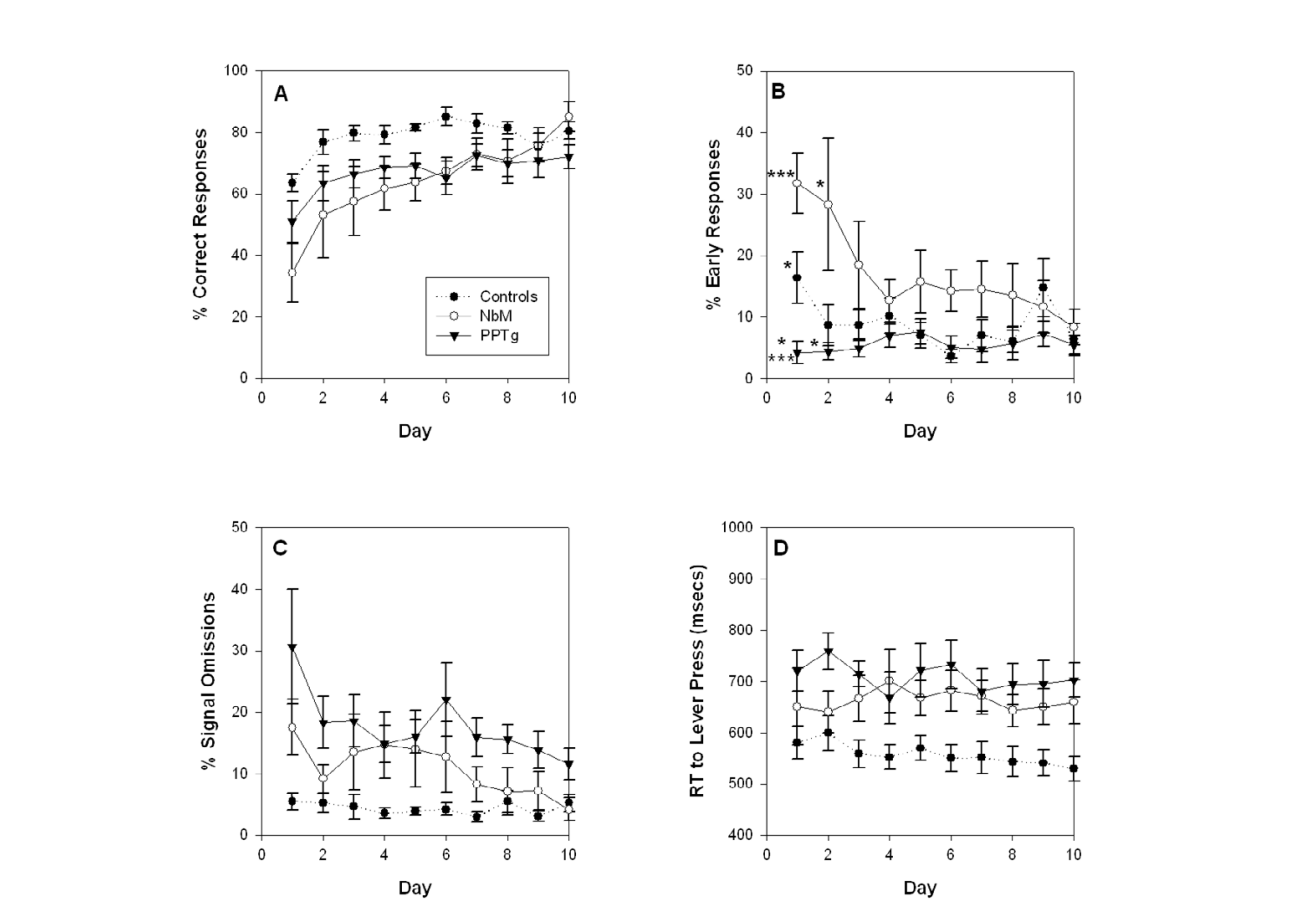
**Mean (+/- SE) performance of lesion and control animals in the sustained attention task over post-surgical days 1–10**. The 4 panels show: A – Percent correct responses (response to bright light), B – Percent early responses (response to dim light), C – Percent signal omissions, D – Latency to lever press on a correct response. Full statistical analysis appears in the text. There were significant effects of day on signal omissions and correct responses suggesting an element of relearning following lesion surgery. PPTg lesioned animals made significantly more signal omissions (p < 0.001) and had significantly longer latencies (p < 0.005) compared to controls. NbM lesioned animals showed reduced correct responses (p < 0.05) and longer latencies (p < 0.05) compared to controls. Significant group × day interactions were found only in relation to early responses and are depicted in panel B: on day 1 there were significant differences between the PPTg group and controls (* p < 0.05) and between PPTg and NBM groups (***p < 0.001); on day 2 the PPTg group were significantly different to the NBM group (*p < 0.05).

Operant data taken over the first 10 days post-surgery (1500 ms signal duration) are illustrated in Figure 2. Consistent with our previous observations [3] there was a significant effect of PPTg lesions on **signal omissions**. However, although Figure 2C suggests that omissions were also increased in the NbM group, this increase was smaller than in PPTg lesioned rats and was not significant. Two-way ANOVA conducted on the percentage of **signal omissions **(2C) confirmed a significant main effect of group (F _2,18 _= 13.08, p < 0.001). Post hoc Tukey tests showed that the PPTg group made significantly more omissions than control animals (p < 0.001) while the NbM group was not significantly different from either the control animals (P = 0.068) or the PPTg lesioned animals (p = 0.063). NbM rats' performance during the first 10 days was therefore somewhere in between normal performance, and the impaired performance of PPTg animals. Overall, there was also a significant main effect of day on signal omissions (F _5.61,100.96 _= 3.42 p = 0.005) suggesting that all groups decreased the number of omissions made throughout the course of days 1–10. The group × day interaction was not statistically significant (F _5.61,100.96 _= 1.06 p = 0.403).

In order to assess whether the behavioural deficits present in both of the lesioned groups were due to cholinergic cell loss we opted to correlate cholinergic cell counts with performance on measures showing significant lesion effects. The mean number of signal omissions made by PPTg lesioned animals across days 1–10, however, did not correlate with the total bilateral count of NADPH diaphorase positive cells in the region of the PPTg r(7) = 0.55; p = 0.20.

The data for **percent correct responses **(2A) indicated that both PPTg and NbM lesion groups performed poorly in relation to the control group. The two-way ANOVA performed on these data showed a main effect of group (F _2,18 _= 4.55 p = 0.025). Post hoc Tukey tests showed that the NbM lesioned animals were significantly different to controls (p = 0.037). The PPTg group were not different to the NbM group (p = 0.928) and only approached the significance level compared to the control group (p = 0.065). There was a significant main effect of day (F _5.30,95.44 _= 11.87, p < 0.001), but no significant group × day interaction. Thus all groups improved performance over time. Pearson correlation showed that the mean number of percent correct responses in NbM lesioned animals did not correlate significantly with ChAT positive cell counts in this structure r(6) = 0.62; p = 0.19.

**Percent incorrect responses **(dark period lever presses; data not illustrated) showed no significant group differences (F _2,18 _= 2.96, p = 0.077). Neither was there a significant group × day interaction suggesting that none of the lesion groups were significantly impaired on this measure (F _6.28,113.02 _= 0.94, p = 0.517). **Percent early responses **(dim period lever presses – 2B) revealed a significant group × day interaction (F _9,162 _= 3.26, p < 0.001). Follow-up restricted one-way ANOVAs found a significant group effect on day 1 (p < 0.001) and 2 (p = 0.026) only. Tukey tests conducted for the day one ANOVA revealed the significant differences to be between the PPTg group and both other groups (controls: p = 0.015, NbM: p < 0.001), indicating that PPTg lesioned rats made significantly less early responses on the first day of post-surgical testing. On day two the PPTg group was different only to the NbM group (p = 0.026).

Finally, data for the **latency to press the lever on a correct response **(2D) also showed a significant effect of group (F _2,18 _= 7.51, p = 0.004). Post hoc Tukey tests confirmed that both lesion groups were significantly different to controls (For NbM: p = 0.046, for PPTg: p = 0.004). Neither the day effect (F _6.14,110.42 _= 0.84, p = 0.541) or the group × day interaction (F _6.14,110.42 _= 0.81, p = 0.640) were significant. Additional Pearson correlations showed that cholinergic cell counts (NADPH diaphorase positive cells in PPTg lesioned animals, and ChAT positive cells in NbM lesioned animals) did not correlate with this deficit. For the PPTg animals: r(7) = 0.51; p = 0.24. For the NbM animals: r(6) = -0.06; p = 0.91.

### Post-surgery sustained attention data: Effect of signal length manipulations

**Figure 3:**
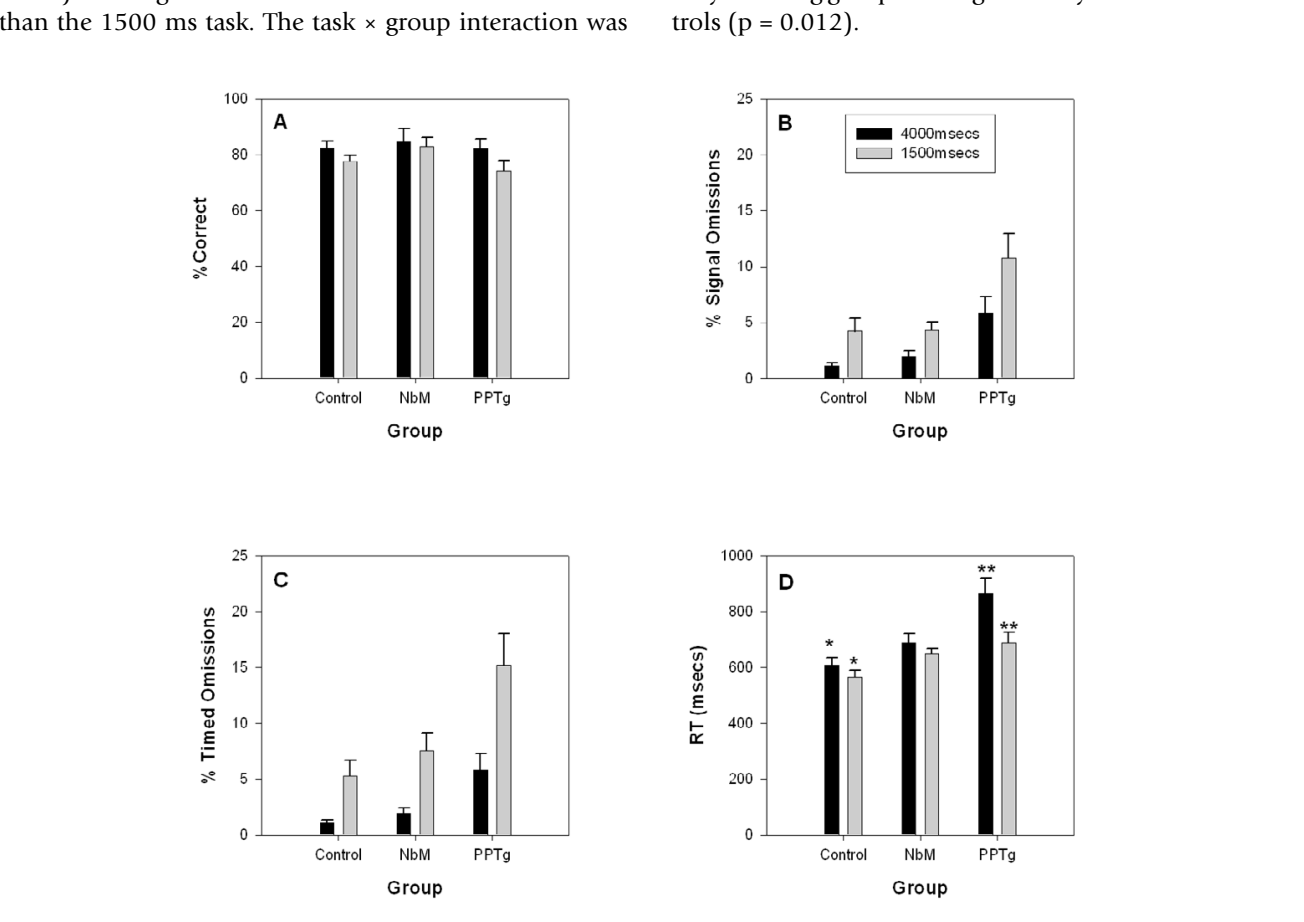
**Mean (+/- SE) performance of lesion and control animals under different signal length conditions**. Data for the 1500 ms signal represent combined data from days 11–15 while data for the 4000 ms signal represent combined data from days 16–20. The 4 panels show: A – Percent correct responses (response to bright light), B – Percent signal omissions (failure to respond during the bright signal), C – Percent timed omissions (failure to respond within 4000 ms of the onset of the bright signal), D – Latency to lever press on a correct response. Full statistical analysis appears in the text. Main effects of signal length (without interaction) were found for timed omissions (p < 0.001), signal omissions (p < 0.001) and percent correct responses (p < 0.001). Significant signal length effects were only present in the PPTg group (p < 0.005) and the controls (p < 0.05) for the latency measure. Significant task × group interactions were found only in relation to the reaction time data depicted in panel B: there were significant differences between the 1500 and 4000 msec conditions in the control group (* p < 0.01) and in the PPTg group (**p < 0.009).

In order to address whether the effect of increasing the signal length on the number of omissions was due to the lengthened visual signal, or due to the lengthened time window in which to make a response, we compared the effect of signal length on both "signal omissions" and "timed omissions" (see Figure 3). "Signal omissions" were calculated exactly as "omissions" in the Kozak et al. [3] study while "timed omissions" were calculated as no response within 4000 ms of signal onset, regardless of the actual signal duration. The **percent signal omissions **(3B) confirmed a significant effect of task (F _1,18 _= 111.96, p < 0.001) with signal omissions lower in the 4000 ms task than the 1500 ms task. The task × group interaction was not significant (F _1,18 _= 0.67, p = 0.52). However, there was a main effect of group (F _2,18 _= 7.99, p = 0.003). Post hoc Tukey tests revealed that the significant difference was between the PPTg and control group only (p = 0.002), indicating that, at this stage of the experiment, the small omissions effect was no longer present in the NbM lesioned rats. However, **timed omissions **(3C) also showed a significant main effect of task (F _1,18 _= 55.65, p < 0.001) with no significant task × group interaction (F _1,18 _= 0.86, p = 0.44). Again this was due to the percentage of omissions being lower in the 4000 ms task than the 1500 ms task. There was also a main effect of group (F _2,18 _= 5.68, p = 0.012) with Tukey tests confirming again that only the PPTg group were significantly different from controls (p = 0.012).

Analysis of **percent correct **data (3A) revealed a significant effect of signal length manipulations on the performance of the groups (F _1,18 _= 22.17, p < 0.001) with percentage correct responses lower in the 1500 ms task than the 4000 ms task. There was no significant group × task interaction (F _1,18 _= 1.77, p = 0.20) suggesting that this effect was the same for all groups. There was not a significant group effect on this measure (F _2,18 _= 0.80, P = 0.47). For **percentage of dark responses **there was a significant main effect of group (F _2,18 _= 5.41, p = 0.014) with Tukey tests revealing that the NbM group made significantly less dark responses from day 11 onwards in comparison to the control group (p = 0.011). Neither the effect of task (p = 0.18), nor the interaction (p = 0.36) was significant. **Percent early responses **showed no significant main effects or interaction: task p = 0.43, task × group p = 0.33, group p = 0.75. Finally, **latency to lever press **on a correct response (3D) revealed a significant task × group interaction (F _1,18 _= 7.08, p = 0.005). Both the task effect (p < 0.001) and the group effect (p = 0.002) were also significant. Paired t-tests to examine the main effect of task in each group showed that both the PPTg (p = 0.009) and the control group (p = 0.010) displayed significant task effects while the NbM group did not (p = 0.269).

### Post-surgery video data: Days 1, 10, 11, 16

**Lever orientation during the dim light **(data not shown) was the only measure from the dim light period to show a significant group effect (F _2,18 _= 4.26, p = 0.031). Posthoc Tukey tests showed that the significant difference was between the PPTg group and the NbM group only (p = 0.035) with the NbM lesioned animals orienting more than PPTg lesioned animals. The group × day interaction was not significant (F _3.14,28.30 _= 0.71, p = 0.560), but there was a significant effect of day (F _1.57,28.30 _= 12.72 p < 0.001): all groups oriented less on day 1 in comparison to the other days. **Houselight orientation during the dim light **showed neither a group effect nor a group × day interaction (F _2,18 _= 3.31, p = 0.060 and F _6,54 _= 1.60, p = 0.166 respectively). The main effect of day was significant (F _3,54 _= 15.13, p < 0.001) resulting from increased houselight orientation on day 1 in all groups in comparison to the other days. **Unconditioned behaviours – rearing, sniffing and grooming – during the dim period **also showed neither a group effect (F _2,18 _= 0.86, p = 0.434) nor a group × day interaction (F _4.38,39.42 _= 1.10, p = 0.373). The day effect was significant (F _2.19,39.42 _= 12.26, p < 0.001), again resulting from increased unconditioned behaviours from all groups on day 1 in comparison to the other days.

**Figure 4:**
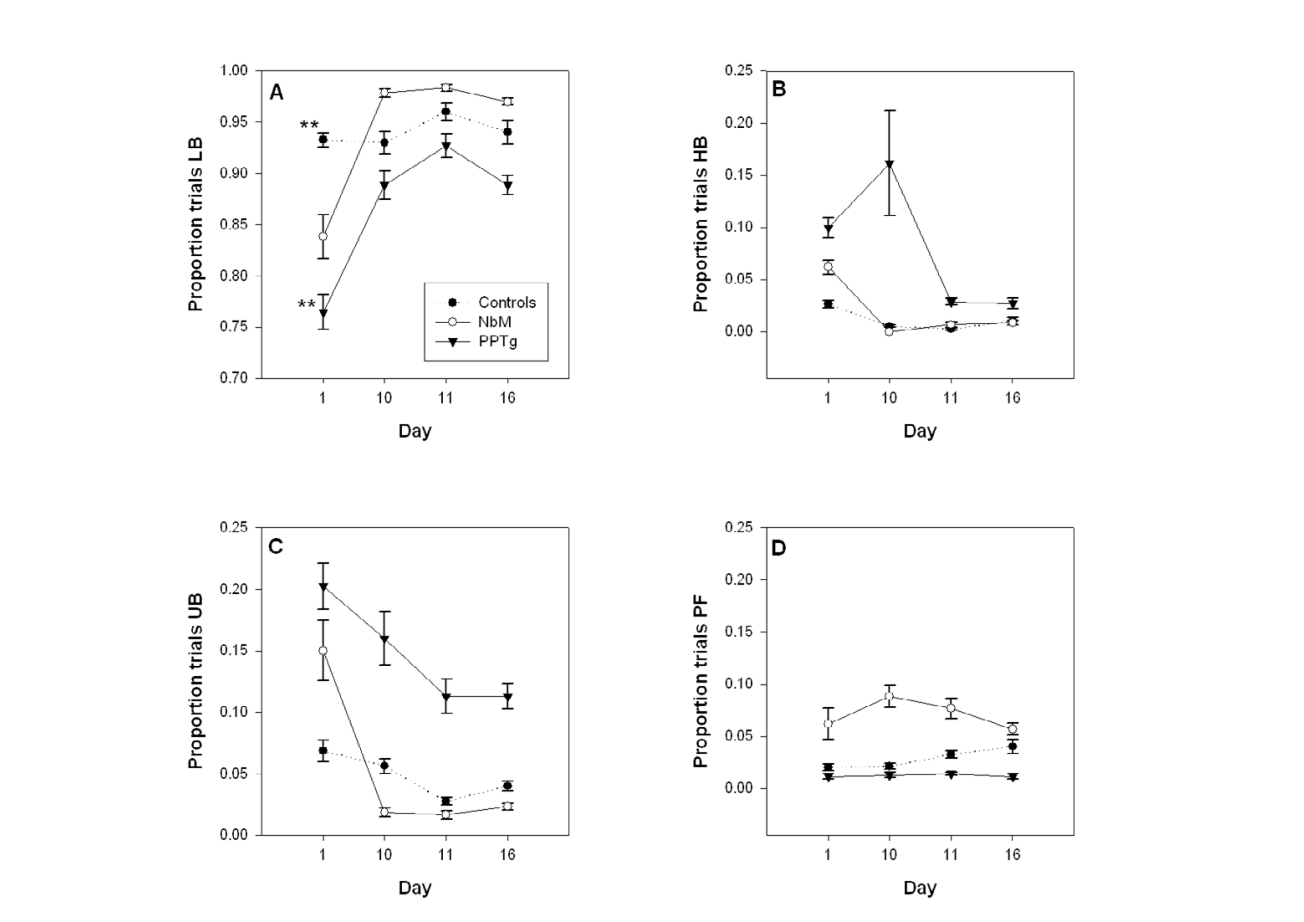
**Mean (+/- SE) proportion of trials on which coded behaviours were observed in the video footage**. Data are taken from days 1, 10, 11, 16. The 4 panels show: A – Bright light lever orientation (LB), B – Bright light houselight orientation (HB), C – Expression of unconditioned behaviours during the bright light (UB), D – Lever pressing failures (PF). Full statistical analysis appears in the text. PPTg lesioned animals had reduced bright lever orientation on day 1 compared to controls (p < 0.01) and showed significantly increased houselight orientation with increased unconditioned behaviours. NbM lesioned animals showed only increased lever pressing failures compared to PPTg lesioned animals (p < 0.05). Only the data for lever orientation to the bright light (panel A) showed a significant group × day interaction. On the first day, there was a significant difference between the PPTg lesioned group and the control group (** p < 0.009).

Data for **lever orientation to the bright light **are shown in Figure 4A. Both the group × day interaction (F _3.86,34.71 _= 3.13, p = 0.02) and the main effect of group (F _2,18 _= 4.23, p = 0.031) achieved statistical significance. The main effect of day was also significant (F _1.93,34.71 _= 13.03, p < 0.001). Follow up restricted one-way ANOVAs were performed to assess the effect of group on each day for the purpose of interpreting the interaction. Only data from day 1 showed a significant group effect: F(2,18) = 5.72, p = 0.012. Post-hoc Tukey tests showed that the significant difference was between the PPTg rats and control rats only (p = 0.009). **Houselight orientation during the bright light **is shown in Figure 4B. For this measure, the main effect of group was significant (F _2,18 _= 6.36, p = 0.008). Post hoc Tukey tests showed that the difference was between the PPTg lesioned rats and the both other groups (controls: p = 0.009, NbM: p = 0.038). There was also a main effect of day on this measure (F _2.02_, 36.35 = 7.67, p = 0.002) with all groups orienting more on day 1 than the other days. The group × day interaction was not significant (F _4.04_, 36.35 = 0.87, p = 0.494). These data also were not correlated with NADPH diaphorase cell counts in PPTg lesioned animals: r(7) = -0.09, p = 0.85. Data for **unconditioned behaviours expressed during the bright light **are shown in Figure 4C. There was a significant main effect of group (F _2,18 _= 6.53, p = 0.007) with post hoc Tukey tests confirming that PPTg lesioned rats expressed more unconditioned behaviours in comparison to controls (p = 0.018) and NbM (p = 0.013). The group × day interaction was not significant (F _6,54 _= 2.00, p = 0.082), but there was a main effect of day (F _3,54 _= 9.10, p < 0.001). Data from Figure 4C suggest that the day effect was a spurious result of increased unconditioned behaviours on Day 1 in NbM lesioned and PPTg lesioned rats, with no day effects apparent in the control group. Again, as previously, these data were not correlated with NADPH diaphorase cell counts in PPTg lesioned animals: r(7) = 0.10; p = 0.83. Finally, data for **lever pressing failures **are shown in Figure 4D. There was a significant main effect of group on this measure (F _2,18 _= 5.64, p = 0.013) with post hoc Tukey tests confirming that it was the NbM lesioned rats that had significantly more press failures than the PPTg lesioned group (p = 0.011). The difference between the controls and the NbM approached the significance level (p = 0.058). There was no group × day interaction (F _5.20,46.81 _= 0.26, p = 0.936) and the day effect was not significant (F _2.60,46.81 _= 1.35, p = 0.270). Lever pressing failures however, did not correlate with cholinergic cell counts (ChAT) in NbM lesioned animals r(6) = -0.33; p = 0.52.

### Histological analysis

**Figure 5:**
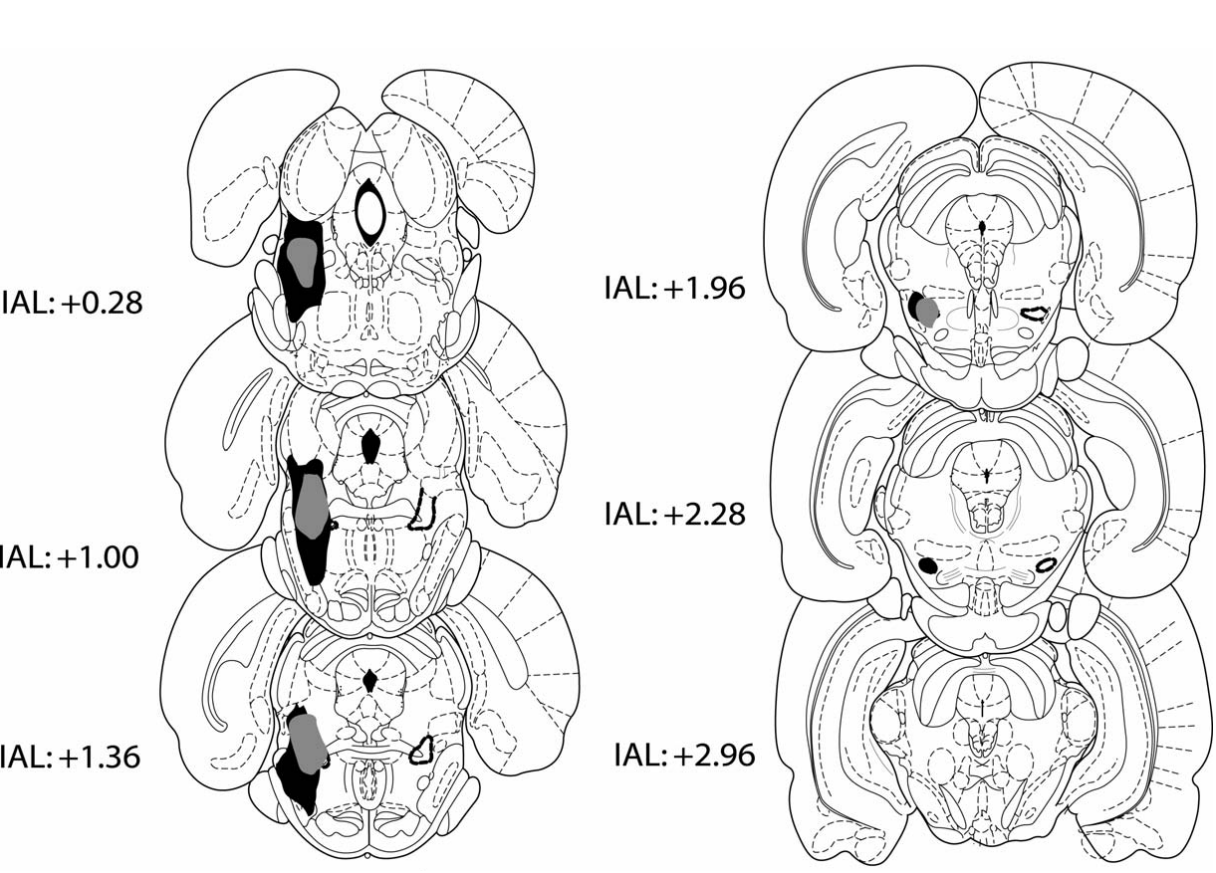
**A representation of ibotenate lesions of the PPTg included in the data analyses**. The largest lesion is shown in black and the smallest in grey. The location of the PPTg is indicated by a dashed black outline on the right of the diagrammatic sections. Distance is given from the interaural line in mm.

**Figure 6:**
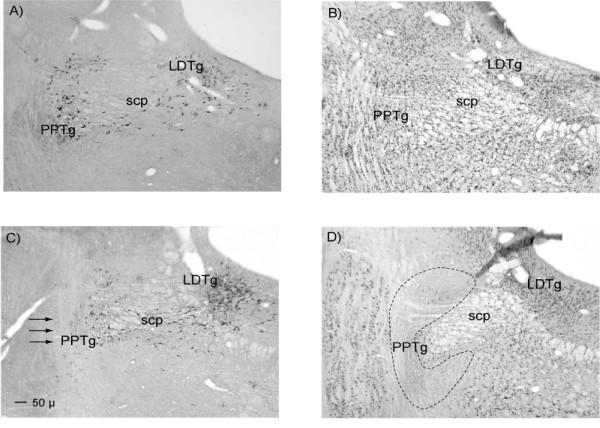
**Photomicrographs illustrating the extent of survival of presumed cholinergic neurons following ibotenate lesions of the PPTg**. The four panels show: A – NADPH diaphorase staining in control tissue, B – NeuN/cresyl violet staining in control tissue, C – NADPH diaphorase staining in lesioned tissue with arrows to indicate the expected location of NADPH positive neurons, D – NeuN/cresyl violet staining in lesioned tissue with the borders of the PPTg indicated by black dashed lines. Abbreviations: PPTg = pedunculopontine tegmental nucleus, LDTg = laterodorsal tegmental nucleus, SCP = superior cerebellar peduncle.

**Figure 7:**
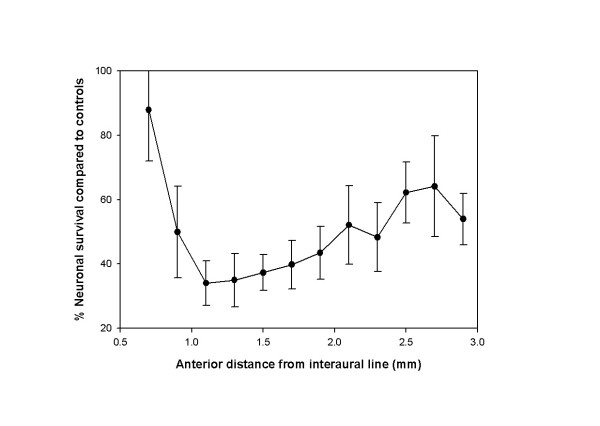
**Graph depicting the degree of survival of NADPH diaphorase positive neurons throughout the rostral caudal extent of the PPTg**. Data are expressed as the mean (+/- SE) percentage neuronal count of control animals.

In total eight rats had to be rejected from the PPTg lesion group. One had poor perfusion preventing histological assessment of lesion location in this animal. Three had placements that were off target or non-symmetrical. In addition to these, two rats had extensive unilateral damage to the superior colliculus and two rats exhibited unusually poor recovery from the surgical procedure (they showed signs of dehydration that did not improve with ip. saline, and were unresponsive in the operant chamber: these rats were humanely killed). In total, seven rats were retained to form the PPTg group in the data analysis. All seven had evidence of neuronal damage centred on the PPTg, and in all cases placement of the lesion was in the caudal region of PPTg with damage extending into some of the more rostral sections. Figure 5 shows a representation of these PPTg lesions. Assessment of NeuN/cresyl violet staining showed large areas of damage in posterior PPTg, but NADPH diaphorase staining revealed pockets of surviving nitric oxide synthase positive neurons (these are presumed ACh neurons). Figure 6 illustrates an example of the survival of these neurons while Figure 7 indicates the percentage of surviving NADPH diaphorase positive neurons throughout the rostro-caudal extent of PPTg.

**Figure 8:**
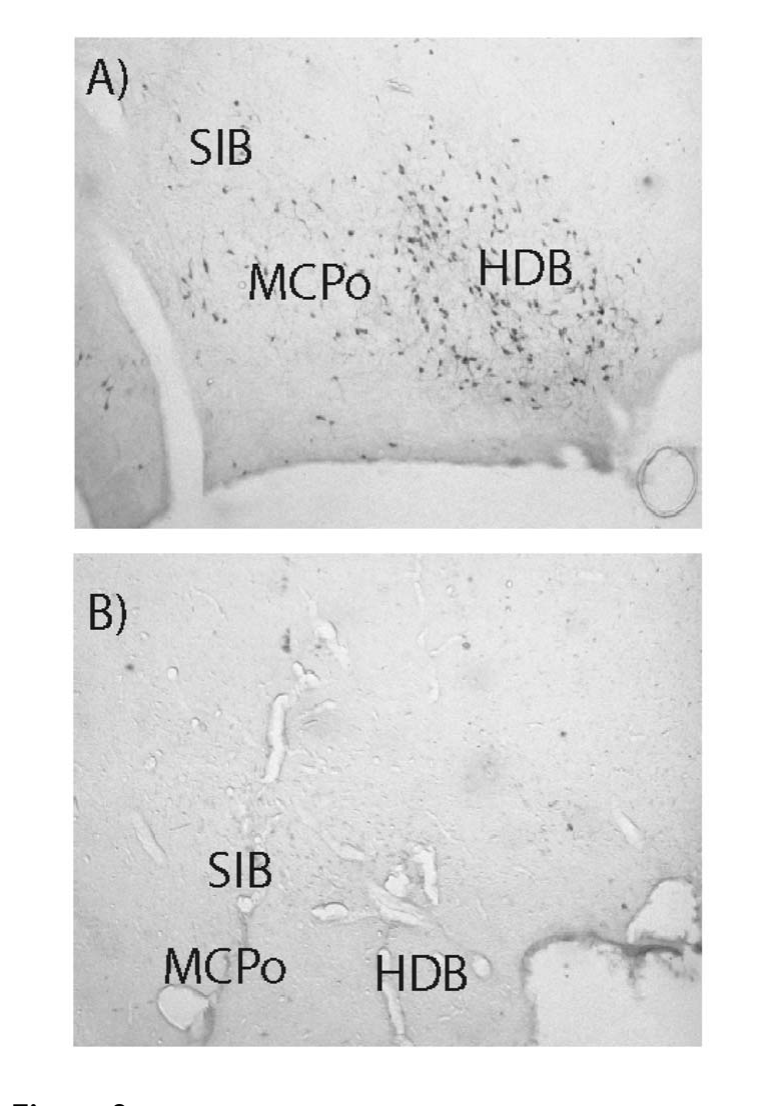
**Photomicrographs illustrating the extent of ChAT positive neuronal loss in the region of the nucleus basalis magnocellularis**. The two panels show: A – ChAT positive neurons in a control animal (infusion of Dulbeccos saline), B – Extensive loss of ChAT positive neurons in the same location in an animal who received bilateral infusions of 192 IgG Saporin. Abbreviations: HDB = Horizontal diagonal band of Broca, MCPo = Magnocellular preoptic nucleus, SIB = Substantia innominata basal part.

**Figure 9:**
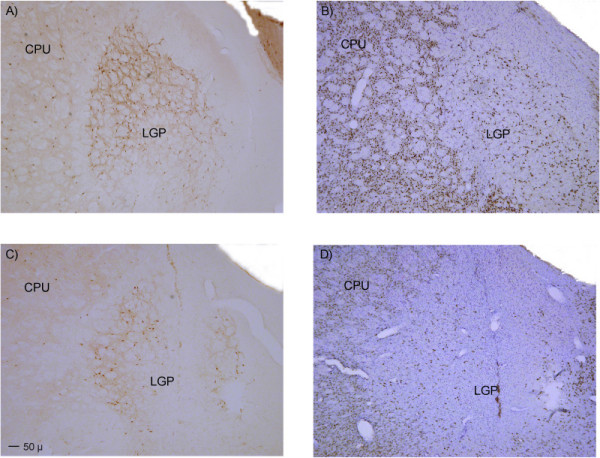
**Photomicrographs illustrating the extent of neuronal damage to the lateral globus pallidus**. The two panels show: A – Loss of parvalbumin immunopositive neurons, B – Loss of NeuN/cresyl violet reactivity in the same area. Abbreviations: LGP = lateral globus pallidus, CPU = caudate putamen.

Three rats were excluded from the NbM lesion group on the basis that the ChAT immunohistochemistry indicated poor loss of cholinergic neurons in the region of the NbM (less than 60%) in at least one hemisphere. The remaining six rats had approximately 60–90% loss of NbM cholinergic neurons in each hemisphere, with less than 50% loss of cholinergic neurons in the medial septum and vertical diagonal band. Representative tissue sections illustrating the loss of ChAT positive staining are presented in Figure 8. Table 1 illustrates the extent of regional loss of cholinergic neurons in each rat included in the data analysis. NeuN/cresyl violet staining revealed a small volume of focal non-selective damage around the site of the infusion in the lateral globus pallidus. The non-selective nature of this damage was confirmed by parvalbumin staining. It was visible in each rat over a rostrocaudal spread of approximately 250 μm. Figure 9 presents an example of this lateral GP damage.

**Table 1:** Loss of cholinergic neurons from the basal forebrain as a percentage of control tissue cell counts.

**Rat No**.	**MS/VDB**	**HDB/MCPO**	**SIB**	**NbM/SI**
**205**	25.35	49.40	73.06	67.29
**207**	47.69	87.01	69.43	88.92
**215**	17.12	36.95	26.42	69.44
**216**	32.01	70.81	54.92	80.65
**217**	17.18	62.76	39.90	70.46
**228**	29.31	67.61	32.64	72.44
**Mean % loss**	32.83	73.83	54.83	86.67

Neurons were identified by ChAT immunohistochemistry. Abbreviations: MS/VDB medial septum/vertical limb of the diagonal band; HDB/MCPO horizontal limb of the diagonal band/magnocellular preoptic area; SIB substantia innominata basal part; NbM/SI nucleus basalis magnocellularis/substantia innominata.

## Discussion

### 192 IgG Saporin Selectivity

Infusions of the selective cholinergic toxin 192 IgG saporin, directed at the NbM, produced significant loss of the corticopetal cholinergic neurons in this region with less than 50% loss of cholinergic neurons projecting to the hippocampus (for example, from the medial septum). Nevertheless, despite the strong selectivity for cholinergic neurons, both NeuN/cresyl violet staining and parvalbumin immunohistochemistry revealed the presence of non-selective damage focal to the infusion site, consistent with at least one previous report [12]. We note, though, that the concentration delivered was almost double that used in our study. While it can be argued that this restricted damage in the lateral globus pallidus was relatively minor in comparison to the extensive loss of the cholinergic basal forebrain neurons, it is nevertheless worth noting its existence. It can be misleading to believe that a neurotoxin has "pure" selective actions when in fact it can have multiple effects. Smaller doses may prove more selective than the dose administered here (0.13 μg per hemisphere) since doses as low as 0.075 μg per hemisphere have been used in attention research [27]. Although the study by McGaughy et al. [27] also assessed the impact of 192 IgG Saporin on parvalbumin-IR neurons in the globus pallidus, and found no damage to this neuronal group, this could reflect the fact that the assessment was of unilateral lesions only. We note also that the dosage used in our current study is not atypical of doses used by other researchers (for example, 0.15 μg per hemisphere [28,29]; 0.125 μg per hemisphere [30]).

### Lesion Induced Behavioural Impairments

The operant data obtained from the sustained attention task showed that both 192 IgG Saporin lesions of the NbM and ibotenate lesions of the PPTg produce impairments on this test of sustained attention. However, the nature of these impairments is qualitatively and quantitatively different. For PPTg lesions the deficit was characterised by an increase in the number of omitted responses and slower correct response latencies. There was also some evidence of a decline in correct responses. In comparison to the findings of Kozak et al. [3], the PPTg lesion impairments observed in this experiment were clearly less severe. Furthermore, Kozak and her colleagues found a significant increase in dark responses that was not present in the current study. These differences, both in the severity and in the nature of the PPTg impairment, most likely reflect the refined lesion technique that was adopted for the current experiment. The use of glass micropipettes allowed us to deliver smaller quantities of toxin in order to create more discrete damage. This technique resulted in damage that did not encompass the more anterior portions of the *pars dissipata *and which also left some surviving "islands" of cholinergic neurons. Therefore, the extent of damage to the PPTg is clearly a factor in determining the strength of the deficit observed in this task (for example, the number of omissions made by PPTg lesioned rats). Qualitative changes in the deficit, however, could be the result of differences in the presence/absence of damage out with the borders of the PPTg. Kozak et al. note that lesions were large and that damage was sustained to other structures [3]. Interestingly the study also noted that the number of dark responses did not change following the manipulation of signal length and so was unlikely to be part of the same impairment that produced more omissions. Given that an additional impairment in dark responses would detract further from the number of correct responses made by PPTg lesioned rats it is not surprising that correct responses were also significantly reduced in that study but not in the present one. Significantly, research assessing PPTg lesioned rats' behaviour in the five choice serial reaction time task has revealed a deficit that precisely matches that found in the current experiment: reduced percent correct responses, increased percent omissions, and slower latency to respond [6]. Furthermore, in Inglis's study anticipatory responding (comparable to "dark" responses in the current task) was not increased in PPTg lesioned animals [6].

### Why do PPTg lesioned rats perform poorly on this task?

Previous work to assess the effects of PPTg lesions in this sustained attention task has demonstrated a stimulus length dependent omissions deficit. However, because of the design of the task, increasing the signal length also increased the time window in which a correct response could be made. Thus, functional explanations other than impaired attention are viable. One alternative hypothesis that can be discounted on the basis of the current results is that PPTg lesioned rats suffer a motor impairment. Both the "signal omissions" measure and the "timed omissions" measure in the current study showed signal length sensitivity, a finding that could suggest that rats make fewer omissions in the 4000 ms task because the visual signal is present for longer (a manipulation of attentional load) and not simply because they have a longer timeframe in which to respond. We do note, however, that "late" responses were not rewarded in the 1500 ms task and so it is possible that rats opted to withhold any slow or delayed responses once the light signal had extinguished. If this were the case, this could also account for the results observed. However, informal observation of PPTg lesioned rats' behaviour in the operant chamber from the video data further supports the absence of a motor deficit. PPTg lesioned rats can be seen moving normally, without any obvious motor difficulties, around the operant chamber and were clearly able to behave exactly as sham lesioned rats on some task trials (see additional file 1 for sham lesioned rats and additional file 2 for PPTg lesioned rats). It is of note that latency to lever press (time between signal onset and lever press) was longer in PPTg lesioned animals since this measurement would incorporate some aspect of movement time as the animals require to locate themselves close to the lever. However, conditioned ambulatory locomotion in PPTg ibotenate lesioned animals is not impaired on the radial maze even when the excitotoxic lesion is larger and more complete than that produced here [31].

Instead, we consider alternative cognitive explanations for the PPTg lesion increase in omissions in this task. Our main hypothesis (as established in the Kozak et al. study [3]) is that PPTg lesioned rats are unable to sustain attention on task relevant stimuli. The operational characterization of this hypothesis could include increased unconditioned behaviours – an increase in grooming, for example, would represent a failure to sustain attention during task performance as it is evidence of PPTg lesioned rats being distracted. However, an increase in the duration and frequency of unconditioned behaviours could also be consistent with a failure of response selection, because the appropriate conditioned response (the lever press) is unable successfully to interrupt the ongoing behaviour that emerges during the ITI. The problem is dissociating these two hypotheses.

Careful analysis of the video data is helpful in this respect. These data demonstrated that PPTg lesioned rats' failure to respond was accompanied by (i) reduced orientation to the lever during the bright light, when they should be pressing the lever; and (ii) an increase both in orientation to the houselight, and in the amount of unconditioned behaviour displayed (including rearing on the side walls, sniffing the cage floor and grooming). A hypothesis of attentional distraction is *not *supported by the increase observed in orientation to the conditioned stimulus (the houselight). The increase in unconditioned behaviour was also limited to the duration of the bright light as no increase was observed during presentation of the dim light. Thus PPTg lesioned rats were only "distracted" during the time that they should be emitting the conditioned lever press response. Such findings favour the hypothesis that PPTg lesioned rats suffered a deficit in the appropriate selection of the conditioned lever press response. We note that this hypothesis is also supported by the results of previous studies on PPTg function [6,32-34]. Interestingly, this conclusion would also fit with the findings of our earlier study [3] if we assume that the dark responses effect was an artifact of damage out with the PPTg. For example the Kozak et al. paper argues that the observation of more than one error type, one in advance of the target stimulus (incorrect responses) and the other after it (omission of a response) indicates that the PPTg deficit was *not *simply in initiating a response once the stimulus had appeared [3].

The proposed functional role for PPTg in the successful performance of goal directed behaviour would be consistent with the ability of cholinergic PPTg neurons to modulate dopamine levels in the nucleus accumbens via connections with the ventral tegmental area [35-39]. Dopamine activation in the nucleus accumbens is associated with cue-controlled operant responding [40,41]. This hypothesis would be directly testable by application of a muscarinic agonist to the ventral tegmental area in PPTg lesioned rats (which would obviously be expected to alleviate the omissions deficit). In addition, a deficit in cue-controlled operant responding following PPTg lesions would be expected to generalise to operant tasks without strong sustained attention demands. However, in this respect it is interesting to note that recent work suggests that the effects of nucleus accumbens dopamine manipulations vary depending on the degree to which cue presentation can be predicted [40]. This factor has been argued to relate to the likelihood that ongoing behaviours are required to be interrupted in order to perform the instrumental response. One crucial aspect of taxing sustained attention is precisely that cue presentation is unpredictable. Sustained attention is often defined as a state of readiness to *detect *and *respond *to certain specified environmental changes that occur rarely and unpredictably. The problem therefore lies in separating signal detection from response selection. Future work at this laboratory will address PPTg lesion deficits in a sustained attention task that clearly separates these two factors. We plan to assess PPTg lesioned rats' performance on an attention task where failure to detect a visual signal requires a lever press response, rather than a response omission [12,42]. In addition, we plan also to assess whether PPTg lesioned rats performance is comparable to the performance of intact rats operating in the presence of a distractor. If we are firmly to discount the hypothesis of attentional distraction in PPTg lesioned rats, we would expect that the characteristics of their impairment would *not *match those of intact rats working under conditions that prevent adequate sustained attention.

### The effects of NbM lesions on this task

It is clear that the 192 IgG saporin lesions of the NbM did affect performance on this task. An unexpected result from the current experiment, however, was that NbM lesioned rats suffered significant lever pressing problems in response to the bright light. This can be easily identified in the video footage (see additional file 3). NbM lesioned rats were observed orienting to the lever in preparation for making a response and, once the bright signal became illuminated, placing their forepaw onto the lever. Two lines of evidence, though, indicated that a press had been unsuccessful. First, the bright light did not extinguish immediately following the rat placing its forepaw onto the lever (the task was programmed for the light to do this immediately following a successful press) and second, the rats rapidly moved away from the lever to nose poke the pellet dispenser tray, suggesting that they were expecting a reward. On finding the dispenser tray empty, those still having time to emit another lever press would attempt to do so (as in the 4000 ms task – see additional file 3). To the best of our knowledge, problems with lever pressing in NbM rats have not been reported previously following 192 IgG saporin lesions. Indeed, video analysis is rarely undertaken in operant tasks. Furthermore, it is interesting to note that these difficulties in executing the lever pressing action could potentially account for the operant effects seen in this task (reduced percent correct responses, increased omissions, slower correct lever press latency). We note however, that the significant lever pressing deficit does persist in NbM lesioned rats long after performance on the operant measures (e.g omissions) has returned to a level comparable with controls. Therefore press failures do not directly equate with response omissions. Perhaps, as time passed over days, NbM lesioned animals learned to overcome their press failures such that some of the trials on which a press failure occurred became correct response trials rather than omissions.

Some form of non-cognitive impairment after NbM lesions might indeed be expected. Previous work using the excitotoxin AMPA (that would damage cholinergic and non-cholinergic neurons) has suggested the presence of forelimb somatosensory deficits in rats with lesions of the NbM [43]. It is, however, questionable whether or not loss of cholinergic neurons from the NbM itself is responsible for the deficits described here since the significant behavioural deficits did not correlate with cholinergic cell counts in the NbM (though group numbers in these analyses were admittedly low). Thus the deficit might be ascribed to: i) the limited non-selective damage to lateral globus pallidus neurons or ii) damage to cholinergic neurons in other basal forebrain regions. It is however worth noting that, as well as the prefrontal cortex, the NbM sends cholinergic projections to the somatosensory cortex. It is not inconceivable, therefore, that loss of cholinergic function here would lead to the type of impairment we have described.

If it is the case that a transient somatosensory impairment is responsible for the deficits seen here after NbM lesions, should we conclude that the NbM has no true role to play in sustained attention? No: it is very clear that in other, more demanding tasks of attention (e.g those where multiple signal lengths are presented in the same session), NbM lesioned rats perform very poorly indeed. What the present data have highlighted is that, in the early post-lesion period, other non-cognitive processes might be operating that could affect performance in tasks with a lever press demand.

## Conclusion

We have studied the effects of ibotenic acid lesions of the PPTg and 192 IgG saporin lesions of the NbM on a sustained attention task. We selected this particular task for use simply because our previous research had shown impaired performance on it following PPTg lesions (increased omissions). However the functional interpretation of this effect in terms of dysfunctional attention was open to alternative accounts such as impaired movement. We took the effects of 192 IgG saporin lesions of the NbM to be a benchmark of impaired attention against which we could qualitatively and quantitatively assess the effects of PPTg lesions, and included video data analysis as a means of determining exactly why PPTg lesioned animals omit trials on this task. Firstly, our results revealed surprising effects of 192 IgG saporin administered to the NbM: impaired lever pressing. However, whether this resulted from the damage sustained to cholinergic neurons, or from the additional non-selective damage focal to the infusion site, cannot be determined. The PPTg lesion results suggest that increased omissions occur in this group due to an increase in unconditioned behaviours such as grooming, sniffing the operant box grid floor, and rearing on the side walls. Although this might be evidence of increased distractibility in these rats we argue that the timing of these behaviours (during the bright signal presentation only) suggests impaired conditioned response selection.

## Methods

### Subjects

32 male Lister Hooded rats (Harlan Olac Ltd., U.K.) were used, weighing 280–320 g at the start of testing and 360–420 g at surgery. Rats were kept in temperature and humidity controlled rooms with lights on a 12 h cycle. Rats were pair housed on arrival in the vivarium but were separated immediately prior to training. They were maintained on a food restriction regime such that they gained weight each week by ~5–10 g. In order to achieve this, 20 g food was given per rat, per day at the end of the day's testing schedule; water was freely available in the home cage throughout. All experiments were conducted with the authority of the appropriate U.K. Home Office Licences and adhered to guidelines set out in the Animals [Scientific Procedures] Act (1986), and the "Principles of laboratory animal care" (NIH publication No. 86-23, revised 1985).

### Behavioral Training and Testing

All training and testing was conducted in Med-Associates operant boxes (Med-Associates, St Albans, Vermont, USA) housed within ventilated light and sound insulated chambers. The front wall of the operant chamber was equipped with a retractable lever and pellet dispenser that delivered 45 mg precision pellets (Noyes Precision Pellets, 45 mg, formula A/1, Sandown Scientific, U.K.) into a food tray. In addition the chamber contained a houselight on the rear wall that could be illuminated at either 3 (dim) or 11 (bright) lux. The equipment was controlled by a PC linked to a Med-Associates interface system. For the first session rats were placed into the operant chamber with one food pellet delivered every 4000 ms until a maximum of 96 pellets had been delivered. During this period the houselight was on but was switched off on retrieval of a pellet. For the next session rats were trained to lever press for food reward on a FR1 schedule with the houselight on. A lever press switched the houselight off and led to a 4000 ms time out prior to it being illuminated again, and the lever activated to record a press. 50 lever presses were required over 2 consecutive days prior to beginning training on the vigilance task. The vigilance task followed a procedure previously used to test frontal lobe function in rats [25,26]. A single trial for this procedure began in darkness (20 sec), followed by dim illumination of the houselight for a random variable duration (5–8 sec). The houselight was then increased to bright illumination for 4 sec during which the rat had to press the lever to obtain a food pellet (a correct response). A task schematic is shown in Figure 1. The session ended once the rat had achieved 50 correct responses, or once it had been in the operant chamber for 60 min, whichever was the sooner. The lever was extended into the box throughout the duration of the testing session. A lever press during the dark period was considered an incorrect response, while a response during the dim illumination was considered an early (anticipatory) response. Failure to respond at all during the trial constituted an omission. Rats were punished for omissions, incorrect and anticipatory responses by 4 sec time out periods in darkness. A lever press during the time out period re-started the time out period.

Following acquisition of this task to a criterion of at least 65% correct and no greater than 20% omissions for 2 consecutive days the bright signal duration was progressively decreased in 500 ms steps until the same criterion was reached at 1500 ms bright signal duration. Training and testing was conducted 5 days a week during the 12 h lights on period. Throughout training and testing behaviour in the operant chambers was observed on monochrome monitors connected to miniature monochrome cameras with infrared LEDs fitted in the operant chambers (Tracksys, Nottingham, U.K.). Video data were recorded using standard VHS video recorders.

### Surgery

Rats were divided into 3 groups, matched by bodyweight. These were 192 IgG saporin lesion of the NbM (N = 9), ibotenate lesion of the PPTg (N = 15), and controls. The control animals received either Dulbeccos saline infusions into the NbM (N = 2), phosphate buffer infusions into the PPTg (N = 3) or were non-operated (N = 3). NbM lesions were made with both hemispheres lesioned in the same surgical session, while the second hemisphere PPTg lesions were made 1 week after the first hemisphere lesions (as previously [3,31,44]). This is because bilateral PPTg excitotoxic lesions made in the same session have been found to produce high mortality rates. All rats received carprofen analgesia ("Rimadyl", Pfizer, Sandwich UK; 0.05 ml s.c. per rat) immediately prior to surgery. Rats were anaesthetized with 1.0 ml/kg sodium pentobarbitone ("Sagatal", Rhône-Mérieux, Harlow UK; 60 mg/ml i.p.; diluted 50:50 with sterile water) and placed in a David Kopf stereotaxic frame with their skull level. In PPTg rats a small craniotomy was performed to reveal the superior sagittal and transverse sinuses and the surface of dura. Dura was cut under microscope guidance at the injection site. PPTg lesions were made using pressure injection via drawn glass micropipettes (30–40 μm tip). In cases where the stereotaxic coordinates placed the micropipette over a blood vessel it was carefully pulled clear using an adapted 30 gauge needle (the tip was bent over to create a miniature hook). The pipettes were left *in situ *for 5 min following infusion. There were 3 × 200 nl infusions of ibotenate (Tocris-Cookson Ltd, Bristol, UK; 0.02 M solution in phosphate buffer [pH 7.4]; final pH adjusted to pH 7.0 using 2 M NaOH) per hemisphere at the following coordinates: 0.2 mm anterior to the interaural line, 2.0 mm lateral to the midline sinus, and 6.2 mm ventral to dura; 0.6 mm anterior to the interaural line, 2.0 mm lateral to the midline sinus, and 6.2 mm ventral to dura; 1.3 mm anterior to the interaural line, 2.1 mm lateral to the midline sinus, and 7.0 mm ventral to dura. NbM lesions were made using a 1.0 μl Hamilton glass syringe with 1 infusion of 0.5 μl 192 IgG saporin (Advanced Targeting Systems, San Diego CA) per hemisphere at a concentration of 0.26 μg/μl in Dulbeccos saline. Infusions were made at the following coordinates: 0.7 mm posterior to bregma, 2.9 mm lateral to the midline sinus and 6.7 mm ventral to dura. 192 IgG saporin infusions were made rapidly to create a bolus and the needle was left *in situ *for 3 min prior to, and 3 min post infusion. NbM lesioned rats had at least 10 days to recover from surgery while PPTg lesioned rats had 7 days to recover from their second surgery prior to beginning post-surgical testing.

### Post-surgical testing

All rats received 20 post-surgical test sessions. For days 1–10 these occurred using the 1500 ms bright duration stimulus. Days 11–15 switched to a 4000 ms bright duration stimulus, and days 16–20 reverted back to the 1500 ms bright duration stimulus. Data collected included the latency to press the lever during a correct response (measured from the onset of the bright light); percentage of correct, incorrect and anticipatory responses; and omissions. The omissions errors were additionally classified into two types: "signal omissions" and "timed omissions". Signal omissions were taken as failure to respond during the bright light, the same type of omission as that recorded as "omissions" previously [3,25,26]. The version of the task used here additionally measured "timed omissions" as a failure to respond within 4000 ms of bright signal onset, regardless of whether the light signal was present for 4000 ms or only for 1500 ms. This measure was taken as an attempt to overcome the motor/attention confound of increasing the signal length because the time frame for responding was the same in both tasks.

In addition to the above operant measures video data were also recorded in order further to analyze rats' behaviour during the task. The main concern with these data was to address why PPTg lesioned rats made more omissions, as well as to compare their behaviour with that of NbM rats. We selectively analysed video data from day 1, 10, 11 and 16. Days 1 and 10 were chosen to enable a comparison of performance at the start of post-surgical testing period with performance after a period of relearning. We aimed to check that any changes in behaviour were stable over this 10 day period. Day 11 was selected because it represents the first day using the longer signal duration (4000 ms) and day 16 because it represents the first day of re-testing at the 1500 ms signal duration. We assessed behaviour at the time of the dim and bright light signals. Behaviour was not assessed during the dark periods because it was difficult to recognize the start of a new trial from a time out period. All measures were scored as the proportion of trials where the behaviour was observed, because the total number of trials varied for each rat and each session. The following activities were recorded: (i) orientation to the lever, with orienting defined as either whole body or head turned towards the lever; (ii) orienting to the houselight on the rear wall (scored as above); (iii) unconditioned behaviours – rearing (both front paws off the cage floor and standing with one or both forepaws on a side wall, or both front paws off the cage floor but neither paw on a wall and not oriented to either the houselight or the lever), sniffing (continuously for > 2 sec) and grooming (with either tongue, forepaws or hindlimbs for > 2 sec). Finally, observations of NbM lesioned rats unexpectedly revealed failure adequately to depress the lever in response to the bright light. As a result the extent of this behaviour was also included as a measure in the video data analysis.

### Histological analysis

Rats were deeply anaesthetised with 0.8 ml sodium pentobarbitone ("Dolethal", Univet Ltd., Bicester, Oxon, U.K.; 200 mg/ml) by i.p. injection. They were perfused transcardially with 0.1 M phosphate buffered saline followed by 4% paraformaldehyde in 0.1 M phosphate buffer at 20 ml/min. Brains were removed and stored in 20% sucrose in 0.1 M phosphate buffer overnight before being processed further. Prior to sectioning on a freezing microtome brains were cut approximately in half in the coronal plane, with the caudal part containing the PPTg and the rostral part the NbM. Parallel sections were taken at 50 μm with 1:4 used for each stain through the PPTg and 1:6 used for each stain through the NbM. Both sets of sections were stained for neuron-specific nuclear protein (NeuN – antibody: Chemicon International, Hampshire, U.K.) and counterstained with cresyl violet. NeuN is found in neurons but not glial cells. Counterstaining with NeuN and cresyl violet renders areas of excitotoxic lesion in particular very clear because the lesion is easily identifiable by the presence of reactive gliosis (highlighted by the cresyl violet staining) and absence of neurons (revealed by the NeuN stain). Sections through the PPTg were also stained using nicotinamide adenine dinucleotide phosphate (NADPH) diaphorase by a modification of the method of Vincent and colleagues [45-47]. NADPH diaphorase reveals nitric oxide synthase positive neurons in the mesopontine tegmentum; these are virtually all cholinergic [45]. NADPH diaphorase was selected for staining rather than choline acetyl transforase (ChAT) since it provides better resolution of neuronal processes in the region of the pedunculopontine tegmental nucleus. NbM sections were additionally stained for choline acetyltransferase immunohistochemistry and the calcium binding protein, parvalbumin, using an avidin biotin method.

All lesions were identified by cell loss and reactive gliosis using a Leitz "Diaplan" microscope. PPTg lesions were mapped by hand onto silhouettes taken from the atlas of Paxinos & Watson [48]. In addition NADPH diaphorase positive neurons of the PPTg, and ChAT positive cells of the basal forebrain were counted using a square grid graticule. For the basal forebrain ChAT cells were counted using the nomenclature of the atlas of Paxinos and Watson [48] into the following groups: medial septum and vertical diagonal band of Broca, horizontal diagonal band of Broca and magnocellular preoptic nucleus, substantia innominata basal part, and nucleus basalis magnocellularis and substantia innominata. Cells were counted through a block of tissue identifiable from 1.20 mm anterior to bregma to 3.30 mm posterior to bregma in the atlas of Paxinos and Watson [48]. NADPH diaphorase positive cells were counted within the estimated borders of the PPTg, excluding cells belonging to the subpeduncular tegmental and laterodorsal tegmental nuclei, as far as these NADPH diaphorase cells were visible rostro-caudally. This ranged from 2.9 mm anterior to the interaural line to 0.7 mm anterior to the interaural line in Paxinos and Watson [48]. The cell counting was not subject to correction because the sections were not serial. Given the cell dimensions, the probability of double counting of individual neurons across the sets of sections (at either 1:4 or 1:6) was low. The numbers of neurons identified was intended to provide an estimate of lesion damage rather than an attempt to determine the absolute numbers of neurons in each structure.

### Data analysis

All data were analyzed using SPSS for Windows (v.15.0). Proportional data were arcsin transformed (x' = 2arcsinvx) according to the methods of Zar [49]. Time data were log10 transformed and all data were subjected to the Huynh-Feldt correction for heterogeneity of variance where this was found to be necessary. In such cases adjusted degrees of freedom are reported. Operant data were analyzed for the last 3 days prior to surgery to check for significant pre-existing group effects. Data were transformed into a mean for these three days and then analysed using one-way between subjects ANOVAs. Post-surgical data was analysed first for days 1–10 to check for the presence and duration of lesion deficits. These analyses were 2 way mixed ANOVAs with days as the within subjects factor and group as the between subjects factor. Post hoc Tukey tests were used to follow-up significant group effects. To address specifically the effect of the signal length manipulations, data for each rat on days 11–20 were collapsed into means for each of the signal lengths, such that the 4000 ms data represented the mean for days 11–15 and the 1500 ms data represented the mean for days 16–20. These data were analyzed as two-way mixed ANOVAs with task (signal length) as the within subjects factor and group as the between subjects factor. Again post hoc Tukey tests were used to follow-up group differences. Video data obtained for the dim light and bright light stages were calculated as proportion of trials on which the behaviour was observed. Data were adjusted so that it was calculated against the number of trials where each stage was reached. For example bright data were expressed as a proportion of total trials minus dark and early responses. These data were analyzed over the selected days as two-way between subjects ANOVAs with post hoc Tukey tests to assess the locus of significant group effects.

## Abbreviations

ACh: Acetylcholine; ChAT – Choline acetyltransferase; ITI: Inter-trial interval; NADPH: Nicotinamide adenine dinucleotide phosphate; NbM: Nucleus basalis magnocellularis; NeuN: Neuron specific nuclear protein; PPTg: Pedunculopontine tegmental nucleus; TRN: Thalamic reticular nucleus.

## Authors' contributions

CLR co-designed the experiment, conducted most of the data acquisition, all of the data analysis and co-wrote the manuscript. MJF participated in data acquisition and care of animals postoperatively. MPL assisted with acquisition and interpretation of histological data. PW conceived of and co-designed the experiment, reviewed all of the data analysis and co-wrote the manuscript. All authors read and approved the final manuscript.

## Supplementary Material

Additional file 1 **Sham lesioned animal (bottom right quadrant) in 1500 ms signal duration attention task**. The signal light (houselight) is situated on the right of the quadrant and the lever and pellet receptacle on the left of the quadrant. The pellet receptacle is illuminated. The video runs for two trials. The first trial starts with the "dim" light signal that can be perceived as a strip of light along the operant box floor. The presentation of the "bright" signal can be perceived as a flash of light. There is a period of darkness before the second trial begins. In both trials the animal successfully presses the lever during the bright signal. Note: the additional lights that can be seen in the corners of the quadrants are infrared lights. Click here for file

Additional file 2 **PPTg lesioned animal (top left quadrant) in 1500 ms signal duration attention task**. The signal light (houselight) is situated on the right of the quadrant and the lever and pellet receptacle on the left of the quadrant. The pellet receptacle is illuminated. The video runs for five trials to illustrate the full pattern of PPTg lesioned animals' behaviour (correctly performed trials interspersed with omissions). Each trial starts in darkness. The "dim" light signal can be perceived as a strip of light along the operant box floor. The presentation of the "bright" signal can be perceived as a flash of light. In the first and third trial the rat performs correctly, pressing the lever in response to the bright light signal. On the second and fifth trial the rat omits a response due to prolonged sniffing. On the fourth trial the rat omits a response due to prolonged houselight orientation. Note: the additional lights that can be seen in the corners of the quadrants are infrared lights. Click here for file

Additional file 3 **NbM lesioned animal (bottom right quadrant) in 4000 ms signal duration attention task**. The signal light (houselight) is situated on the right of the quadrant and the lever and pellet receptacle on the left of the quadrant. The pellet receptacle is illuminated. The video runs for one trial. The trial starts in darkness. The "dim" light signal can be perceived in the video as a strip of light along the operant box floor. The presentation of the "bright" signal can be perceived as a flash of light. The chosen trial illustrates failed lever pressing where the forepaw is seen extended onto the lever but the bright light has not extinguished. We have chosen the longer signal duration to illustrate the rat attempting to retrieve a food pellet and returning to the lever for additional presses. Note: the additional lights that can be seen in the corners of the quadrants are infrared lights. Click here for file
